# Supplementary material for: Leveraging Medical Knowledge Graphs Into Large Language Models for Diagnosis Prediction: Design and Application Study
Source: JMIR AI. 2025 Feb 24;4:e58670. doi: 10.2196/58670 (PMC11894347; doi:10.2196/58670)
Supplement: Multimedia Appendix 1 [file ai_v4i1e58670_app1.docx]

**Supplementary Materials**

Data Preprocessing

Here we describe how to extract ground truth CUIs for the CUI prediction training from a given input medical narrative and the target plan sections. We employ two medical concept extractors that rely on UMLS SNOMED CUIs: cTAKES, a Java toolkit [31] and QuickUMLS, a lightweight Python package [32]. For our IN-HOUSE dataset, we utilize cTAKES for note pre-processing, as it is integrated into a pre-built pipeline specifically designed for data analytics within the hospital’s HIPAA-compliant network. On the other hand, for the MIMIC-III dataset, we opt for QuickUMLS due to its user-friendly nature, facilitating the reproducibility of the study on the public dataset. Both cTAKES and QuickUMLS demonstrate similar levels of performance. Table [6](#_bookmark81) presents an example list of diagnoses and CUIs, after being parsed by cTAKES and QuickUMLS. On QuickUMLS, we set the overlapping criteria as score, with jaccard similarity and threshold of 0.9. When running cTAKES v 4.0.0.1, we employed the runPiperFile strategy for our workflow. We set up a PiperFile with the following algorithms to parse out Concept Unique Identifiers (CUIs) from the text: the cTAKES 4.0.0.1 DefaultTokenizerPipeline tokenizer, which included a SentenceDetector, SimpleSegmentAnnotator, and an implementation of the PennTreeBank n-gram tokenizer (TokenizerAnnotatorPTB); the ContextDependentTokenizerAnnotator, which added annotations such as date, time, and measurements, along with POS tagging; the ChunkerSubPipe, which is an implementation of the OpenNLP Chunker; the DefaultJCasTermAnnotator, an implementation of the FastDictionaryLookup that locates token matches and creates concepts; and the classifier AttributeCleartkSubPipe, an implementation of the ClearTK Library.

In order to create the ground truth for the CUI prediction task, we aim to identify the set of CUIs within the plan sections that ensure the existence of the shortest path connecting them to the input CUIs. It is important to note that without such a path, a knowledge graph-based solution will be unable to locate the corresponding CUIs. Mathematically, the ground truth set of CUIs is defined as follows: For every CUI ci in the ground truth set, there exists a path with a maximum length of 2 to the CUI cj, where cj is an element of the input set of CUIs. We exclude paths with lengths exceeding 2 (i.e., more than 2 hops) from consideration, as our empirical findings suggest that source and target concepts connected by 3-hop paths have a lower likelihood of being clinically meaningful.

In practice, for every pair of CUIs (ci, cj), where ci is in the plan sections CUIs and cj in the input text, we run a Dijkstra algorithm to find if there exists a path with length as 2 or less. Algorithm [2](#_bookmark85) presents the workflow of identifying the ground truth CUIs for CUI prediction tasks.

**Algorithm 2** Extract Path-based CUIs as Ground Truth for CUI Prediction Task

1: **procedure** FINDSHORTESTPATHS (PlanSectionCUIs, InputCUIs)

2: Initialize TargetOutput as an empty set

3: **for each** concept *ci* **in** PlanSectionCUIs **do**

4: **for each** concept *cj* **in** InputCUIs **do**

5: Run Dijkstra’s algorithm to find the shortest paths from *ci* to *cj*

6: Let *shortestPath* be the shortest path found, *l* be the length of the *shortestPath*

7: **if** *l ≤* 2 **then**

8: Include *ci* in TargetOutput

9: **else**

10: Ignore *ci*

11: **end if**

12: **end for**

13: **end for**

14: **return** TargetOutput

15: **end procedure**

DR.KNOWs Training Details

#### Training Setup

**Algorithm 1** DR.KNOWS Graph Model Training for Path Selection

**Require:** UMLS knowledge graph *G*, input patient text **x**

1: Extract CUIs from **x**, and build *V*_src_ and *E*_src_ given **x**

2: Initialise CUI embedding CUI(**v***_i_*) as **h***_i_* and relation embedding **e***_t_* as one-hot embedding

3: **while** not halting **do**

4: **if** *t >* 1 **then** Update *E*_src_ by *V*_src_

5: **end if**

6: Update node representation of **v***_i_* based on Eq. [1](#_bookmark16)

7: Generate path embedding **p***_i_* in the Path Encoder. Rank path embedding using MultiAttn or TriAttn to generate attention score *S_i_* for each path

8: Assign new *V*_src_ <- Argmax_N_(*S*).

9:**end while**

10: Calculate loss function based on *V*_src_

11: Update model’s parameter ***θ***

Algorithm 1 was developed to train DR.KNOWS in selecting paths within a KG, employing a Hop-Encode-Rank process. Initially, upon receiving input text, Concept Unique Identifiers (CUIs) are extracted. These CUIs, along with their corresponding relations in the UMLS, are used to construct *V*_src_ and *E*_src_, representing the initial nodes and the edges for the first hop, respectively. The process continues with graph traversal, proceeding one hop at a time towards potential candidate nodes. At this juncture, path encoding and ranking are performed, utilizing attention scores derived from the implemented attention mechanisms (either MultiAttn or TriAttn). The *N* nodes receiving the highest attention scores are then identified as the new starting points (*V*_src_) for the subsequent iteration. Upon reaching the maximum depth *t* during traversal, the process is halted, and we calculate the loss function using the selected nodes *V*_src_, which represent the endpoints of the graph traversals.

We used SapBERT as an encoder to encode input patient text and CUI concepts. The relations (edges) are encoded as one-hot embedding. Within the graph model components in Figure [3,](#_bookmark14) we use a Transformer as the path encoder, and MultiAttn or TriAttn is used as the path ranker. The number of *t* is 2. At each hop iteration during training, the selected top *N* is 8. Then *N* is set as 4 or 6 for inference. The AdamW optimizer with 1e-5 learning rate is used by decaying it every 3 epochs during training. The graph model is trained for 10 epochs with early stopping mechanism to prevent overfitting.

Prompt Engineering Using ChatGPT

We provide manual task-specific prompts in Table [5](#_bookmark23) to ChatGPT, and ask it to paraphrase those prompts by giving an instruction as “Paraphrase this prompt <Manual Prompts> to 50 different prompts”. Then BETTERPROMPT [37] is used to evaluate those prompts providing perplexity, and top 5 prompts with the lowest perplexity are selected for further finetuning. Table [8](#_bookmark91) shows the full list of top 5 "Non-Subject-Matter" and "Subject-Matter Prompts" paraphrased prompts with the corresponding perplexity scores.

We employ the same prompt selection approach as previously stated, that is, to pick the prompt with the lowest perplexity. ChatGPT is sensitive to the prompt. Following the originally selected prompt, ChatGPT does not separate its diagnosis and reasoning generation, as the original prompt does not include specific output requirements. Consequently, we initially evaluate the prompt using 73 samples from the PROBSUM development set. Subsequently, we proceed with the zero-shot experiments on the test set, while making additional adjustments to the prompt to enable ChatGPT to generate diagnoses and reasoning separately. Specifically, we ask ChatGPT to use “<Reasoning>” to separate its diagnoses and assumption. We utilize the same development set to fine-tune the hyperparameters essential for text generation. To ensure consistency and reproducibility, we set the temperature to 1 and impose a maximum limit of 160 tokens for the generated output. This limit prevents excessive generation and the introduction of extraneous information or noise.

Another key observation during the setup of the zero-shot prompt-based experiment is that ChatGPT solely copies the output from knowledge paths without considering the input note. This is because the prompt does not explicitly require ChatGPT to utilize information from the input note. This deviation from our original intention to test if path prompts improve the note summarization task of foundation models undermines the primary focus on the note as the main source of information. Therefore, we add additional instructions to prompt ChatGPT to focus on the input note and be aware of the noisy knowledge graphs.

Table 4 presented the full list of non-subject-matter prompts and subject-matter prompts and their paraphrased prompts with perplexity scores.

T5 Finetuning

In the process of fine-tuning T5 using path prompts, we select the prompt with the lowest perplexity from both the non-subject matter (n=3) and subject matter prompts (n=2). This selection is based on the results shown in Table 4. We utilize the graph model to generate paths for all samples in the training, development, and test sets. These paths will be concatenated with the prompts. Subsequently, we proceed to fine-tune the chosen prompt using the ProbSum training set. As there are four variants of the graph model, we obtain 20 sets of path prompts by combining five prompt styles with the four graph model variants. In the context of path-less prompt-finetuning, we utilize two sets of prompts: one is the non-subject matter prompt, which starts with the "summarize:," (the default setting for T5's training). The other is the subject matter prompt with a slight modification that removes the contents about paths, as we show in Table 4. We then fine-tune T5 with prompts on the ProbSum training set. Three T5-Large variants are finetuned for 20 epochs with early stopping mechanism. The AdamW optimizer with learning rate 1e-5 is the same as graph model training.

| Manual Prompts | Top 5 Paraphrased Variants | Perplexity |
| --- | --- | --- |

|  | Employ these facts to bolster your understanding: | 2.95e-8 |
| --- | --- | --- |
|  | Employ these facts to strengthen your research: | 3.02e-8 |
| You may utilize these facts: | Employ these facts to broaden your knowledge: | 3.47e-8 |
|  | Use these facts to complement your findings: | 3.59e-8 |
|  | Use these facts to broaden your perspective: | 5.10e-8 |

|  | Consider these facts as potentially practical: | 2.26e-9 |
| --- | --- | --- |
|  | Consider these facts as potentially insightful: | 3.50e-9 |
| You may find these facts helpful: | Consider these facts as potentially beneficial: | 4.66e-9 |
|  | Consider these facts as potentially valuable: | 4.68e-9 |
|  | You might find these facts advantageous: | 4.72e-9 |

| Act as a medical doctor, and list the  top three direct and indirect diagnoses from the Assessment. Explain the reasoning and assumptions behind your answer. | Imagine yourself as a physician and outline the three key  direct and indirect diagnoses identified in the Assessment. Discuss the reasoning and assumptions that guide your re- sponse. | 3.98e-4 |
| --- | --- | --- |
|  | In the role of a physician, generate a list of the Assessment’s  three primary direct and indirect diagnoses. Shed light on the reasoning and assumptions that inform your decision. | 5.50e-4 |
|  | Act as a medical doctor and detail the top three direct and  indirect diagnoses resulting from the Assessment. Justify your choices by explaining the reasoning and assumptions involved. | 5.81e-4 |
|  | In your role as a medical doctor, please outline the primary  three direct and indirect diagnoses based on the Assessment. Elucidate the reasoning and assumptions underlying your choices. | 5.83e-4 |
|  | Take on the role of a doctor and indicate the Assessment’s top  three direct and indirect diagnoses. Justify your choices by explaining the underlying reasoning and assumptions. | 6.08e-4 |

| Act as a medical doctor, and list the  top three direct and indirect diagnoses from the input note. You will be provided with some hints from a knowledge graph. Explain the reasoning and assumptions behind your answer. | Imagine you are a medical professional equipped with a  knowledge graph, and generate the top three direct and indi- rect diagnoses from the input note. | 3.47e-4 |
| --- | --- | --- |
|  | Imagine you are a physician incorporating knowledge graph  hints, and outline the top three direct and indirect diagnoses stemming from the input note. Discuss the reasoning and assumptions that inform your diagnostic analysis. | 4.69e-4 |
|  | Act as a doctor, leveraging insights from a knowledge graph,  and compile a list of the primary direct and indirect diagnoses derived from the input note. Explain the reasoning and as- sumptions underlying your responses. | 1.04e-3 |
|  | In the capacity of a physician, employ the information from  a knowledge graph to identify the three principal direct and indirect diagnoses stemming from the input note. Justify your answers by explaining the reasoning and assumptions involved. | 1.10e-3 |
|  | Imagine yourself as a doctor utilizing a knowledge graph, and  provide a comprehensive breakdown of the top three direct and indirect diagnoses resulting from the input note. Clarify the reasoning and assumptions that underpin your diagnostic selections. | 1.17e-3 |

Table 4: The full list of top 4 "Non-Subject-Matter" and "Subject-Matter Prompts" paraphrased prompts with the corresponding perplexity
